# Removal of Ionic Dyes by Nanofiber Membrane Functionalized with Chitosan and Egg White Proteins: Membrane Preparation and Adsorption Efficiency

**DOI:** 10.3390/membranes12010063

**Published:** 2022-01-01

**Authors:** Yue-Sheng Chen, Chien Wei Ooi, Pau Loke Show, Boon Chin Hoe, Wai Siong Chai, Chen-Yaw Chiu, Steven S.-S. Wang, Yu-Kaung Chang

**Affiliations:** 1 Department of Chemical Engineering, Graduate School of Biochemical Engineering, Ming Chi University of Technology, New Taipei City 24301, Taiwan; a0955873266@o365.mcut.edu.tw (Y.-S.C.); chenyaw@gmail.com (C.-Y.C.); 2 Chemical Engineering Discipline and Advanced Engineering Platform, School of Engineering, Monash University Malaysia, Jalan Lagoon Selatan, Bandar Sunway 47500, Malaysia; ooi.chien.wei@monash.edu (C.W.O.); Hoe.BoonChin@monash.edu (B.C.H.); 3 Department of Chemical and Environmental Engineering, Faculty of Engineering, University of Nottingham Malaysia Campus, Jalan Broga, Semenyih 43500, Malaysia; Paul.oke.Show@nottingham.edu.my (P.L.S.); Cloudcws@gmail.com (W.S.C.); 4 Department of Chemical Engineering, National Taiwan University, Taipei 10617, Taiwan

**Keywords:** protein modified nanofiber membrane, chitosan, egg white proteins, removal, cationic and anionic dyes

## Abstract

Electrospun polyacrylonitrile (PAN) nanofiber membrane was functionalized with chitosan and proteins for use in the treatment of dye-containing wastewater. The PAN nanofiber membrane was subjected to alkaline hydrolysis, before being grafted with chitosan and subsequently the proteins from chicken egg white. The resultant nanofiber membrane (P-COOH-CS-CEW) was comprehensively characterized using thermogravimetric analysis, Fourier-transform infrared spectroscopy, and scanning electron microscopy. The efficiency of P-COOH-CS-CEW in removing cationic dye toluidine blue O (TBO) and anionic dye acid orange 7 (AO7) in aqueous solution was evaluated. Based on the performance of model fitting, Langmuir and pseudo-second-order kinetic model could be used to describe the performance of P-COOH-CS-CEW in the removal of TBO (pH 10) and AO7 (pH 2) from the dye solutions. The adsorbed TBO and AO7 dyes can be completely desorbed by an elution solution made of 50% (v/v) ethanol and 1 M sodium chloride. After five consecutive adsorption-desorption cycles, the efficiency of dye removal by P-COOH-CS-CEW was maintained above 97%.

## 1. Introduction

The dye-contaminated wastewater produced from textile, papermaking, printing, and related industries can cause significant environmental issues [1,2]. Due to the complex and robust chemical structures, dyes are very stable in water and are resistant to degradation by light, chemical, or biological treatment. Therefore, removing the dyes using traditional treatment of wastewater can be challenging [3]. The conventional methods used in the dye removal process include biological treatment, coagulation/flocculation, ozone treatment, chemical oxidation, membrane filtration, photocatalytic degradation, and adsorption [4,5,6,7,8,9]. However, these methods are energy-demanding and involve multiple operations, thereby limiting their wide adoption in industrial applications [2]. Adsorption is a popular approach to dye wastewater treatment due to its cost-effectiveness and simplicity of operation [10,11,12,13,14,15]. Sugar cane bagasse, cotton waste, chitin, chitosan, peat, microorganisms (fungus and yeasts), maize cob [16], hazelnut shell, saw dust (derived from walnut, cherry, oak, pitch pine, and pine), cane pitch [17], soy meal hull, banana pitch, Brazilian pine-fruit shell [18,19], and rice husk [20], aqai stalks [21] have all been widely used as adsorbent for dye removal.

Nanofibers are a promising adsorber because of their high ratio of surface area to weight, good flexibility, high porosity, superior mechanical properties and water permeability, and the easiness of surface modification [22,23,24,25,26,27]. These nanofibers were successfully used in the filtration processes (dye removal) that could treat a large amount of wastewater in a short time [22,28,29,30,31,32]. Polyacrylonitrile (PAN) yarn is an inexpensive polymer that can be electrospun into nanofibers. PAN nanofiber membrane is known to possess strong mechanical strength and high chemical stability. Moreover, the nitrile groups (-CN) in PAN nanofibers could be converted carboxyl groups (-COOH) via alkaline hydrolysis and acid neutralization. The -COOH groups on the surface of acidic PAN nanofiber membrane (P-COOH) serve as the sites for the coupling of other functional molecules, such as chitosan. Chitosan is an excellent bio-adsorbent with amino and/or hydroxyl groups that act as the active adsorption sites for capturing cationic and/or anionic dyes [33,34]. Hence, the coupling of chitosan on the P-COOH will boost the functionality of the resultant nanofiber membrane (P-COOH-CS) for the treatment of dye-containing wastewater.

The adsorption capacity of chitosan-derived adsorbents could be improved by functionalization using cross-linking reagents or incorporation of functional groups onto its chitosan backbone [35]. Proteins are a promising functional group that can be coupled to the chitosan backbone or P-COOH. The amino (-NH_2_) and -COOH groups in protein can be easily coupled to either the -NH_2_ group on P-COOH-CS or -COOH group on P-COOH to form P-COOH-CS-CEW and P-COOH-CEW, respectively. Depending on the availability of positively and negatively charged groups, P-COOH-CS-CEW can treat waste products with positive or negative charge, such as heavy metal ions, cationic or anionic dyes in wastewater. Overdue egg white waste can be a good source of proteins for the functionalization of P-COOH-CS. Chicken egg white (CEW) is often used as a source of lysozyme in the studies of protein purification [36,37,38]. Aside from lysozyme, CEW contains other proteins such as ovalbumin (54%), ovotransferrin (12%), and ovomucin (11%) [39].

Acid Orange 7 (AO7) and Toluidine Blue O (TBO) dye are widely used as a mediator for various biochemical reactions as well as a coloring agent for fabrics [10]. To the best of our knowledge, there is no relevant report demonstrating the application of P-COOH-CS-CEW for the removal of AO7 and TBO dyes from aqueous solutions. This study aims to synthesize and discover the potential of a promising protein-functionalized nanofiber membrane in dye removal (AO7 and TBO). P-COOH-CS-CEW was characterized using thermogravimetric analysis (TGA), Fourier-transform infrared (FTIR) spectroscopy, and scanning electron microscope (SEM) analyses. Moreover, the adsorption isotherms and kinetics parameters were investigated. The effects of operating parameters (i.e., initial pH and contact time) on the performance of AO7 and TBO adsorption were also investigated. In addition, equilibrium isotherm and kinetic binding mechanism, dye desorption, and reusability of P-COOH-CS-CEW were studied.

## 2. Materials and Methods

### 2.1. Materials

PAN yarn (*Mw*: 120,000, 93% acrylonitrile, 7% vinyl acetate) and polyethylene terephthalate (PET) were supplied by Fortune Industries Inc. (New Taipei City, Taiwan), while spunbond fabric (basis weight 15 g/m^2^, thickness 90 μm, fiber diameter 300–500 μm) was purchased from Freudenberg Far Eastern Spunweb Co., Ltd. (New Taipei City, Taiwan). CEW, TBO (*Mw*: 305.83 g/mol)**,** AO7 (*Mw*: 350.32 g/mol)**,** 4-morpholineethanesulfonic acid (MES), 1-ethyl-3-(3-dimethylaminopropyl) carbodiimide (EDC) and N-hydroxysuccinimide (NHS) were bought from Sigma-Aldrich (St. Louis, MO, USA). Chitosan (*Mw*: 50,000 g/mol) was acquired from Chengli Industrial Co., Ltd. (New Taipei City, Taiwan). Electrospinning equipment was purchased from Falco Enterprise Co., Ltd. (New Taipei City, Taiwan).

### 2.2. Preparation and Modification of PAN Nanofiber Membranes

A syringe with no. 21 stainless steel nozzle was filled with 10 mL of PAN solution. The PAN solution was prepared by dissolving 1.5 g of PAN yarn in 10 mL of dimethylacetamide (DMAc) at 100 °C. The PAN solution was ejected from the syringe to form nanofibers on a grounded stainless-steel roller pre-wrapped with non-woven PET. The electrospinning process was run for 10 h and the operating conditions were based on the methodology reported previously [38]. The resultant PAN nanofiber membrane was then hot-pressed at 100 °C for 1 h. A heat-pressing treatment enhances the adherence of the nanofiber layer to the PET supporting matrix, and results in a more uniform pore size distribution and a smaller pore size.

Next, the alkaline hydrolysis of PAN nanofiber membrane (25 mm diameter) was conducted in 5 mL of NaOH solution (3 M) at 80 °C for 18 min. The excess NaOH in the hydrolyzed nanofiber membrane was subsequently removed by water washing. Later, the membrane was treated with 0.1 M of HCl to generate the ion-exchange PAN nanofiber membrane, which was abbreviated as P-COOH. The surface density of the carboxyl groups on the P-COOH was determined using TBO dye interaction at pH 10 [40].

Next, a piece of carboxylated PAN nanofiber membrane (P-COOH) was submerged into 5 mL of MES hydrate solution (0.1 M, pH 5) containing EDC (300 μmol), NHS (300 μmol), and chitosan (1 mg/mL). The shaking incubation of the membrane was set at 100 rpm and 25 °C for 3 h. Finally, the chitosan-coupled P-COOH (labelled as P-COOH-CS) was rinsed with distilled water to eliminate any remaining chitosan. The clean P-COOH-CS was dried in an oven at 60 °C. The density of amino groups on the surface of P-COOH-CS was determined using an established protocol reported elsewhere [32].

**Figure 1:**
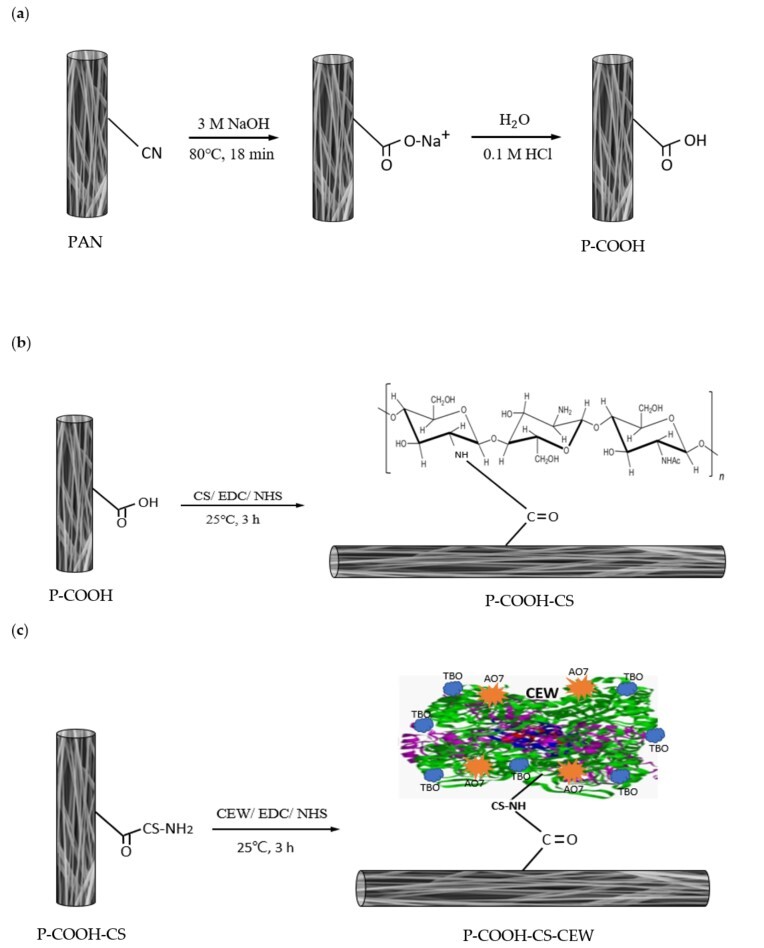
Preparation and modification of PAN nanofiber membranes: (**a**) P-COOH, (**b**) P-COOH-CS, and (**c**) P-COOH-CS-CEW.

To couple proteins on P-COOH-CS, a piece of P-COOH-CS (~0.03 g) was first immersed in 5 mL of MES buffer solution (pH 5, 0.1 M) containing 300 μmol EDC, 300 μmol NHS, and 5 mg CEW. The nanofiber membrane was reacted in the solution for 3 h at 100 rpm, 25 °C, followed by the washing with water and the drying in oven at 60 °C. The synthesized nanofiber membrane was designated as PCOOH-CS-CEW. Figure 1 shows the synthesis routes of carboxylated PAN nanofiber membranes (P-COOH-CS and P-COOH-CS-CEW).

### 2.3. Characterization of Nanofiber Membranes

The morphology and diameter of nanofiber mats were observed using a SEM (S-2600H/EDX, Hitachi, Tokyo, Japan). Before the imaging, all samples were sputter-coated with platinum. The FTIR spectra of samples were acquired using FTIR spectrophotometer (Spectrum One 55366, Perkin Elmer, Akron, OH, USA) with a resolution of 1 cm^−1^ over the wavenumber range of 400-4000 cm^−1^. TGA was carried out using a thermogravimetric analyzer (TGA Q600, Mettle Toledo, Columbus, OH, USA) at a heating rate of 20 °C/min under nitrogen purging. 

The concentrations of AO7 and TBO dyes in the solution were determined using a UV-Vis spectrophotometer (Ultrospec 3100 Pro, GE Healthcare Biosciences, Uppsala, Sweden) at 485 nm and 633 nm, respectively [2,41]. The negatively charged AO7 dye interacted with the positively charged surface functional group (–NH_3_^+^), while the positively charged TBO dye reacted with the negative surface ions (–COO^−^). Both reactions occurred at a stoichiometric ratio of 1:1 [37,38]. The contents of amine and carboxylic group in the nanofiber membranes were then determined. The mechanical properties of the PAN and P-COOH-CS-CEW nanofiber membranes were determined at room temperature using a universal test machine (Tinius Olsen H1KS, SDL ATLAS, Rock Hill, SC, USA). The extension rate of the test was set to 10 mm/min. From the stress–strain curves, the averages of ultimate tensile strength (*TS*, MPa) Young’s modulus (*YM*, MPa), and elongation at break (*EB*, %) were determined.

### 2.4. Effect of Operating Parameters on the Adsorption of Dye Molecules

Similar procedures have been reported for coupling pH and concentration of CEW on the membranes [42,43]. The effects of coupling pH and loaded amount of CEW in the reaction solution on the immobilized density of CEW were investigated. Moreover, the performance of the P-COOH-CS-CEW nanofiber membrane for adsorption of AO7 and TBO dyes was investigated under different pH conditions [42,43].

### 2.5. Equilibrium Isotherm and Kinetic of Dye Adsorption

In the isothermal adsorption studies, P-COOH-CS-CEW nanofiber membranes were first incubated in 50 mL flasks containing dye solutions with different concentrations of AO7 (pH 2) or TBO (pH 10). The nanofiber membranes and dye solutions were mixed at 150 rpm and 25 °C. After 3 h, the residual concentration of dye in the aqueous solution was measured. Then, the equilibrium binding capacity of nanofiber membrane (mg dye/g) was determined. The kinetic of dye adsorption was evaluated using P-COOH-CS-CEW incubated in dye solution (1–3 mg/mL of dye) at 100 rpm and 25 °C for a period of 0–3 h.

### 2.6. Desorption Studies

A piece of P-COOH-CS-CEW (i.d. 25 mm, ~0.03 g) and 5 mL of dye solution (AO7 or TBO; 3 mg/mL) were added to a 50-mL flask. The adsorption process was performed at 25 °C and 150 rpm for 2 h. After that, the membrane was removed from the dye solution and was subjected to washing by buffer solution (pH 2 for AO7, or pH 10 for TBO) to remove the residual and unbound dyes on the membrane. Next, the desorption process was conducted at 150 rpm for 2 h using various types of eluents (5 mL), including 1 M of salts [sodium chloride (NaCl), ammonium sulfate (NH_4_)_2_SO_4_, or potassium thiocyanate (KSCN)] dissolved in 50% organic solvents (ethanol, ethylene, or glycerol). The amounts of eluted dyes in the solution before and after the desorption were recorded. Later, the dye-desorption efficiency was calculated by dividing the amount of desorbed dye by the amount of dye adsorbed on the P-COOH-CS-CEW. In every adsorption–desorption cycle, the adsorption capacity of P-COOH-CS-CEW for the dyes and the elution efficiency (%) were calculated.

## 3. Results and Discussion

### 3.1. Characterization of Nanofiber Membranes

**Figure 2:**
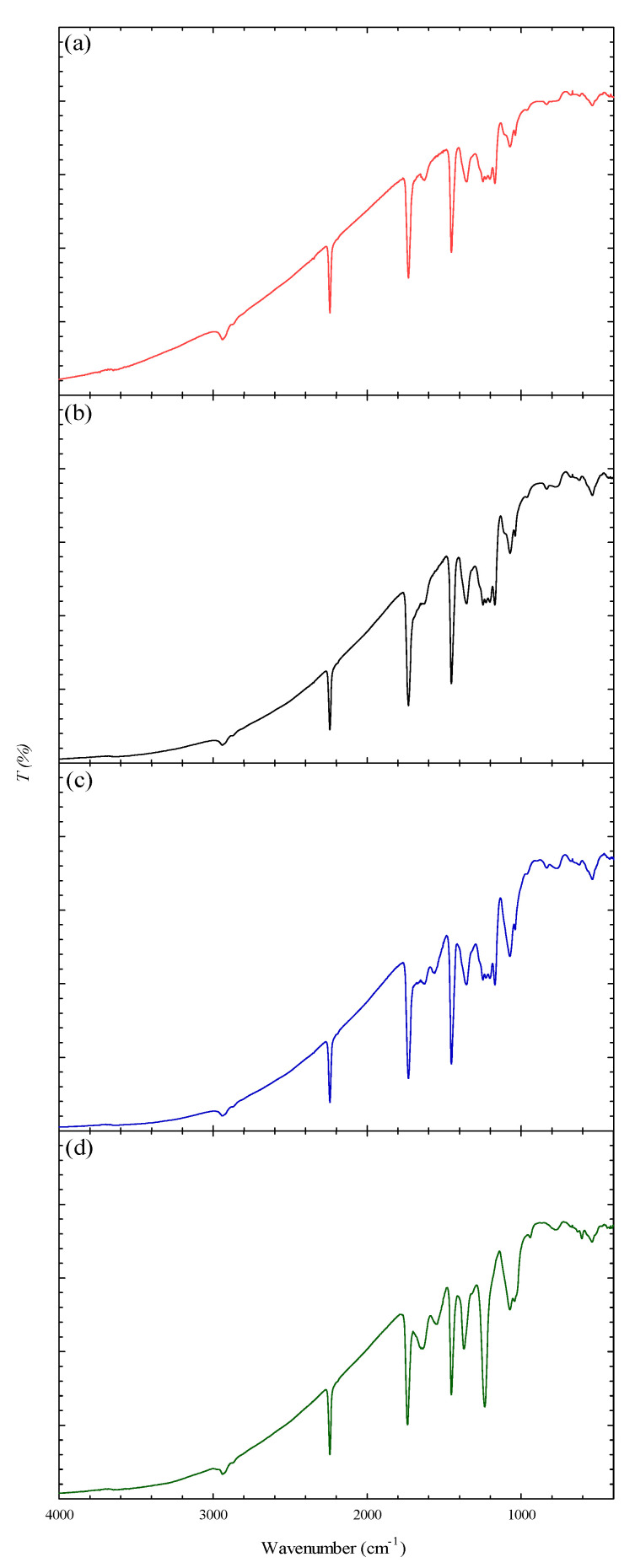
FTIR spectra of (**a**) PAN, (**b**) P-COOH, (**c**) P-COOH-CS, (**d**) P-COOH-CS-CEW.

Figure 2 displays the FTIR spectra of PAN and the modified nanofiber membranes, in which the peaks were observed at 2927 cm^−1^ (C-H bond), 2253 cm^−1^ (-C≡N bond), 1735 cm^−1^ (-C=O bond), and 1452 cm^−1^ (bending vibration of C–H bond) [44]. The prominent peak at 1690 cm^−1^ (-C=O) as shown in the FTIR spectrum of P-COOH indicates the presence of the carbonyl group. In P-COOH-CS, the existence of amide functional group was proven by the presence of a prominent peak at 1245 cm^−1^. P-COOH-CS-CEW’s FTIR spectra showed peaks at 1653 cm^−1^ (Amide I), 1540 cm^−1^ (Amide II), and 1245 cm^−1^ (Amide III), which are associated with amide vibration [14] and C–O vibration (1330–1050 cm^−1^). The presence of C-H and C-OH bonds was proven by the peaks (500–1000 cm^−1^).

**Figure 3:**
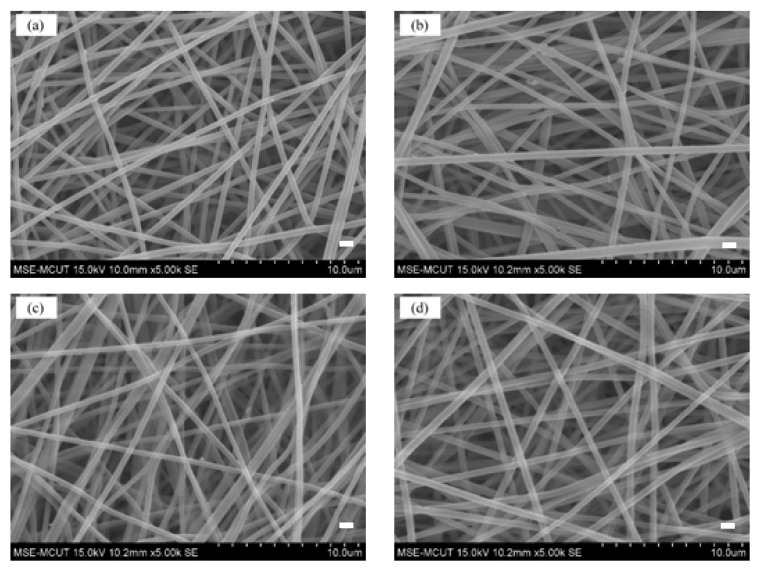
SEM images of (**a**) PAN, (**b**) P-COOH, (**c**) P-COOH-CS, and (**d**) P-COOH-CS-CEW.

The shapes and structures of nanofiber membranes (PAN, P-COOH, P-COOH-CS, and P-COOH-CS-CEW) are shown in the SEM images (Figure 3). For all the fabricated nanofiber membranes, the nanofibers were randomly oriented and in the size range of 400–600 nm. The P-COOH film contained a rough structure. After the surface modification of P-COOH with chitosan and CEW, the thickness of nanofibers of P-COOH-CS-CEW increased marginally. Nevertheless, no obvious change in the overall nanofiber shape was observed. In addition, the surface of the nanofiber membrane modified with CEW protein is partially opaque, showing the presence of charge.

**Figure 4:**
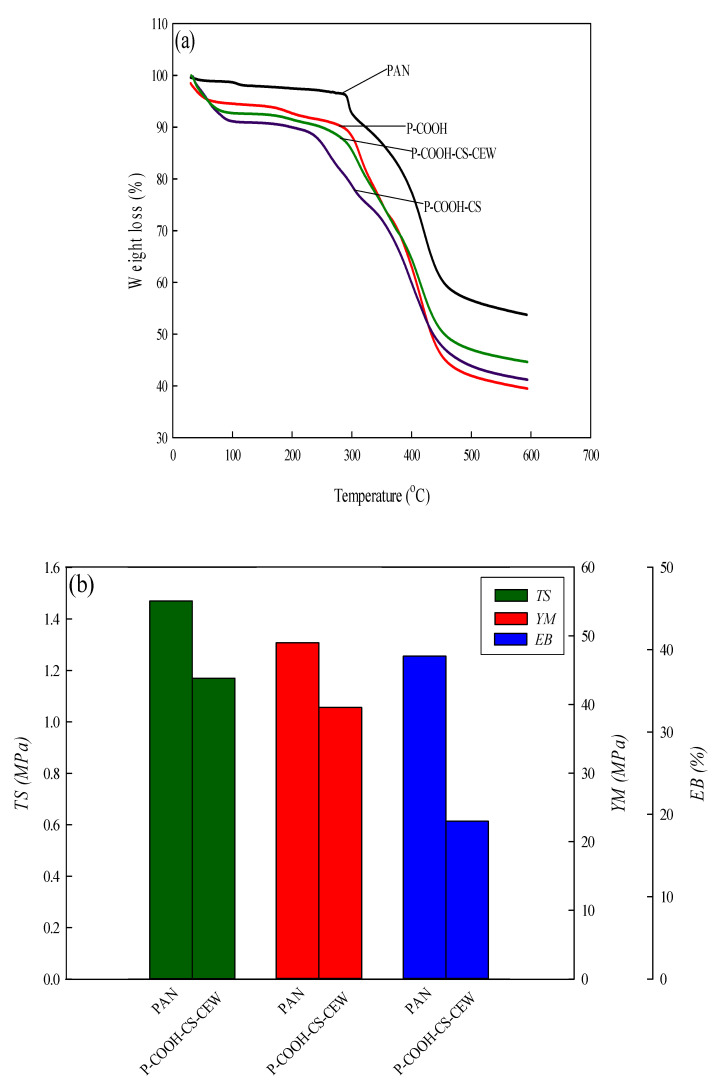
(**a**) TGA curves of PAN, P-COOH, P-COOH-CS, and P-COOH-CS-CEW. (**b**) Mechanical properties of PAN and P-COOH-CS-CEW samples: *TS*, *YM*, and *EB*.

TGA curves of nanofiber membranes are shown in Figure 4a. At 100 °C, a slight weight loss was observed owing to the loss of moisture from the nanofiber membrane. At around 300 °C, these four types of nanofiber membranes exhibited different degrees of weight loss. From 300 °C to 600 °C, the percentages of weight loss in P-COOH, P-COOH-CS-CEW, PAN, and P-COOH-CS nanofiber membranes are 48.73%, 40.86%, 38.92%, and 37.50%, respectively. A higher weight loss was detected in P-COOH-CS-CEW because the thermal stability of the three-dimensional protein-modified nanofiber membrane is weaker.

The mechanical properties (*TS*, *YM*, and *EB*) of the PAN and P-COOH-CS-CEW nanofiber membranes are shown in Figure 4b. The results showed that the mechanical properties of the PAN-COOH-CS-CEW nanofiber membrane are worse than that of the PAN nanofiber membrane, which is the result of changes in the fiber structure of PAN after multi-step processing. In the filtration process, the application of these materials can be hindered by the poor mechanical strength of the membranes. The poor mechanical strength of the nanofiber membranes is mainly due to their high porosity and the weak bond between random fiber orientation and fiber connections. A unique lamination method can be performed on a supporting layer (e.g., polypropylene/polyethylene biocomponents) to increase the mechanical strength of the nanofibers. The treated membranes showed a significant increase in *TS* and *YM*, while maintaining their high porosity and water permeability. Due to their high permeability and mechanical strength, nanofibrous membrane is an excellent for wastewater treatment.

### 3.2. Effect of Coupling pH and Loaded Concentration of CEW on Dye Adsorption

**Figure 5:**
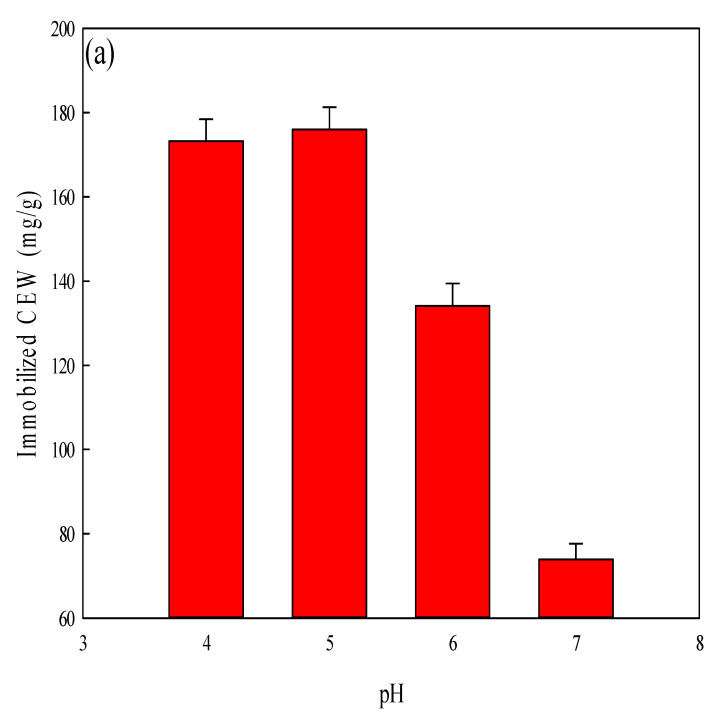

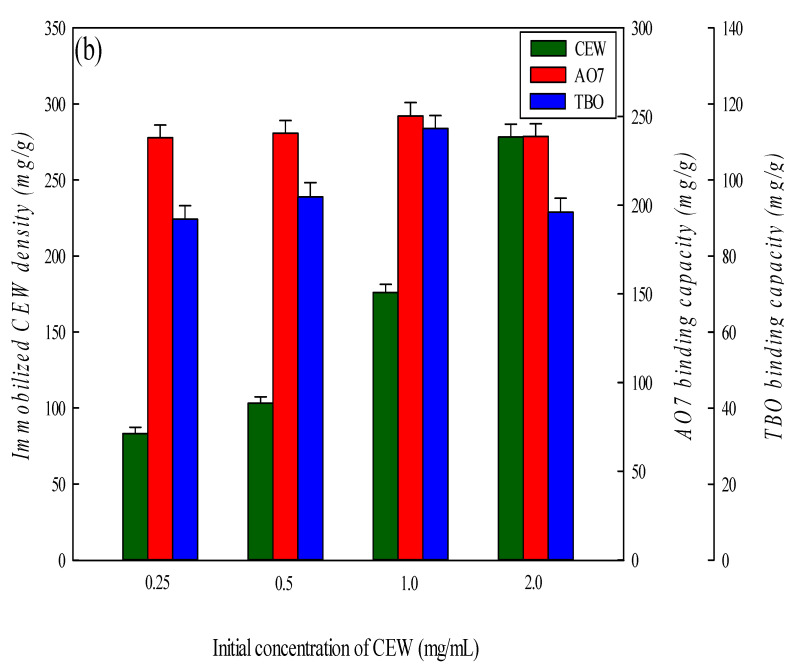
(**a**) Effect of coupling pH on the amount of CEW (1.0 mg/mL) immobilized onto the P-COOH-CS nanofiber membrane. (**b**) Effect of loaded CEW concentration on the CEW immobilization density on P-COOH-CS-CEW nanofiber membrane and the TBO binding capacity. Operating conditions: P-COOH-CS-CEW nanofiber membrane (~0.03 g), CEW concentration (0.25–2.0 mg/mL, 5 mL, pH 5, 3 h, 100 rpm).

The pH used in the coupling of CEW to the P-COOH-CS nanofiber membrane may affect the amount of CEW proteins immobilized on the membrane. In this work, the coupling reaction pH was investigated between 4 and 7, and the results of immobilized density of CEW on the membranes are shown in Figure 5a. The immobilization density of CEW increased from 173.21 mg/g to 176.91 mg/g when the coupling pH was adjusted from 4 to 5. However, the immobilization density decreased to 73.92 mg/g. As a result, a coupling pH of 5 was selected for the subsequent studies involving the immobilization of CEW proteins.

The effect of loaded concentration of CEW (0.25–2.0 mg/mL, 5 mL) on the immobilized density of CEW on the P-COOH-CS-CEW nanofiber membrane was investigated, and the results of immobilized density of CEW on the membranes are given in Figure 5b. By increasing the amount of CEW in the reaction solution from 0.25 mg CEW/mL to 2.0 mg CEW/mL, the binding capacity for AO7 and TBO dyes reached the maximum value of 1.0 mg CEW/mL. However, at the CEW concentration beyond 1.0 mg/mL, the immobilization density decreased to 238.78 mg/g for AO7 and 91.56 mg/g for TBO at a loaded concentration of 0.25 mg CEW/mL. The binding capacity of the P-COOH-CS-CEW nanofiber membrane for AO7 and TBO dyes was decreased by the loaded CEW concentration greater than 1.0 mg/mL. The reduction in binding capacity may be due to the effect of steric hindrance. Hence, the loaded concentration of CEW in the synthesis of P-COOH-CS-CEW nanofiber membrane was fixed at 1.0 mg/mL.

### 3.3. Effect of pH on Dye Adsorption

**Figure 6:**
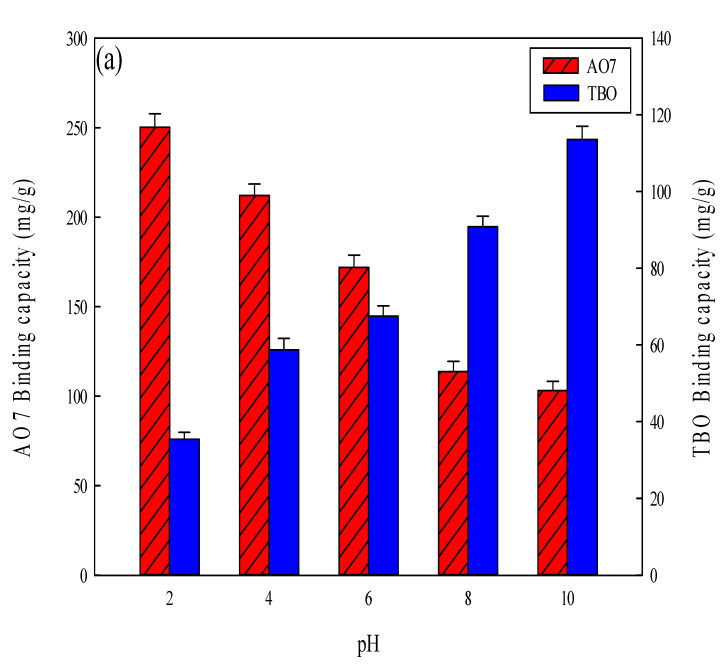

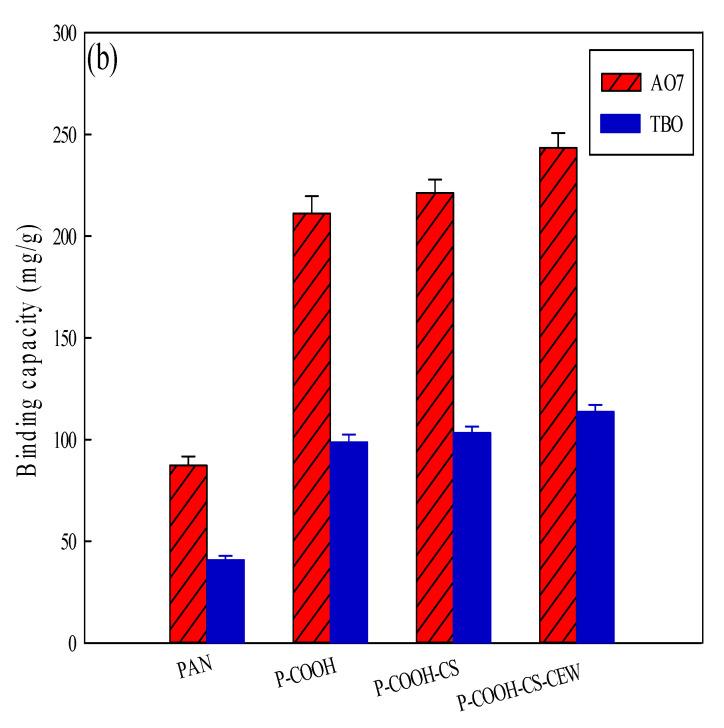
(**a**) Effect of buffer pH on the adsorption of AO7 and TBO on P-COOH-CS-CEW nanofiber membrane. (**b**) A comparison of binding capacity of different nanofiber membranes for AO7 and TBO dyes. Operating conditions: ~0.03 g of nanofiber membranes incubated in 5 mL of dye solution [1 mg/mL of AO7 (pH 2) or TBO (pH 10)] at 150 rpm and 25 °C for 3 h.

In an acidic environment (e.g., pH 2), the electrostatic interaction between the anionic (i.e., sulfonate) group of AO7 and the cationic group on the nanofiber membranes could be governed by the residual ammonium groups (-NH_3_^+^) either on the chitosan or on the CEW proteins. Conversely, in an alkaline environment (e.g., pH 10), the interaction between cationic (i.e., ammonium) group on TBO dye and anionic group (carboxyl) on the nanofiber membranes could be facilitated by the residual acidic group (-COO^−^) either on the P-COOH or on the CEW proteins. The results are shown in Figure 6a. Previous studies have shown that the negatively charged AO7 dye has a higher binding affinity toward the positively charged membrane in an acidic solution (pH 2), while the positively charged TBO dye shows a higher binding affinity toward the negatively charged membranes in an alkaline solution (pH 10) [2,37,40,45]. Hence, in this work, the optimal pH for the adsorption of AO7 and TBO dyes are pH 2 and pH 10, respectively.

Different nanofiber membranes (i.e., PAN, P-COOH, P-COOH-CS, and P-COOH-CS-CEW) were investigated to determine their binding efficiency of AO7 (pH 2) and TBO (pH 10) dyes (1 mg/mL, 5 mL, 25 °C). The binding efficiencies of nanofiber membranes are shown in Figure 6b. The decreasing order of the binding efficiency is given as: P-COOH-CS-CEW (AO7 250.35 mg/g, TBO 113.56 mg/g) > P-COOH-CS (AO7 240.28 mg/g, TBO 103.19 mg/g) > P-COOH (AO7 106.67 mg/g, TBO 98.50 mg/g) > PAN (71.31 mg/g, 40.71 mg/g). The ability of these nanofiber membranes to bind the dyes may be dependent on the hydrophobic, hydrophilic, and ionic interactions between dye and membrane. Moreover, the charge properties of the membrane are affected by the adsorption pH. At pH 2, the membrane carries mostly positive charges, whereas at pH 10, it carries mainly negative charges. Since AO7 and TBO dyes contain positive and negative charges, respectively, the functionalized nanofiber membranes containing macromolecules (e.g., P-COOH-CS and P-COOH-CS-CEW) have a larger binding capacity for AO7 and TBO dyes. Therefore, the macromolecules (e.g., chitosan and protein molecules) coupled to the nanofiber membranes can enhance their binding capacity for the dyes.

**Figure 7:**
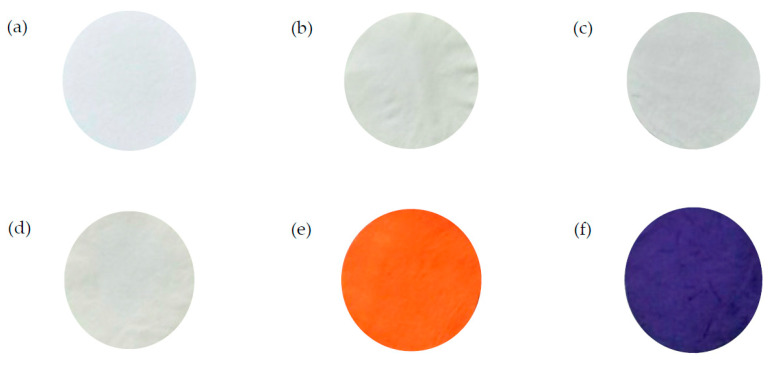
Photos of nanofiber membranes: (**a**) PAN, (**b**) P-COOH, (**c**) P-COOH-CS, (**d**) P-COOH-CS-CEW, (**e**) P-COOH-CS-CEW treated with AO7, and (**f**) P-COOH-CS-CEW treated with TBO.

The photos of nanofiber membrane are shown in Figure 7. After treatment with AO7 and TBO, P-COOH-CS-CEW nanofiber membranes were stained in dense orange and purple, respectively.

### 3.4. Equilibrium Isotherm Studies

Protein molecules immobilized on the P-COOH-CS-CEW have multifunctional groups with different levels of charge density or hydrophobicity. Therefore, dye molecules could be bound to these multifunctional groups via various interactions (e.g., hydrophobic and/or electrostatic interactions). Under alkaline adsorption conditions (pH 10 for TBO dye), egg white proteins may be negatively charged [39]. Therefore, the bonding could be formed between the positively charged group of TBO dye and negatively charged -COOH group of P-COOH-CS-CEW. At pH 2, the adsorption could be potentially due to the bonding between negatively charged group of the AO7 dye and the positively charged ammonium group (-NH_3_^+^) of P-COOH-CS-CEW. Hence, it is suggested that the electrostatic interactions between these functional groups are the main forces for the adsorption of AO7 and TBO dyes by P-COOH-CS-CEW.

**Figure 8:**
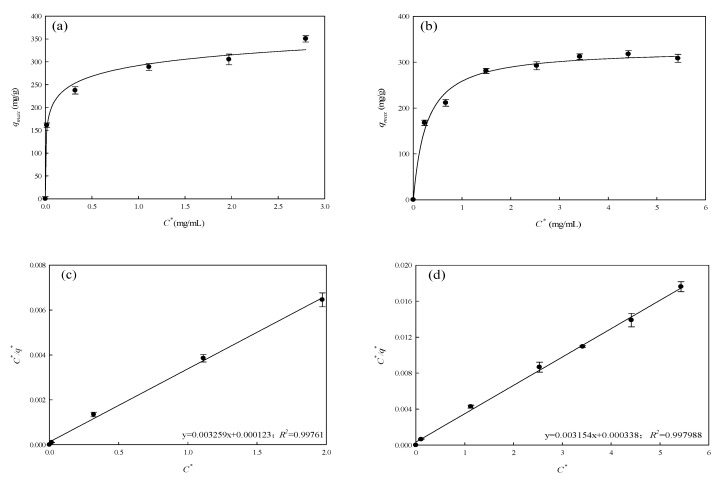

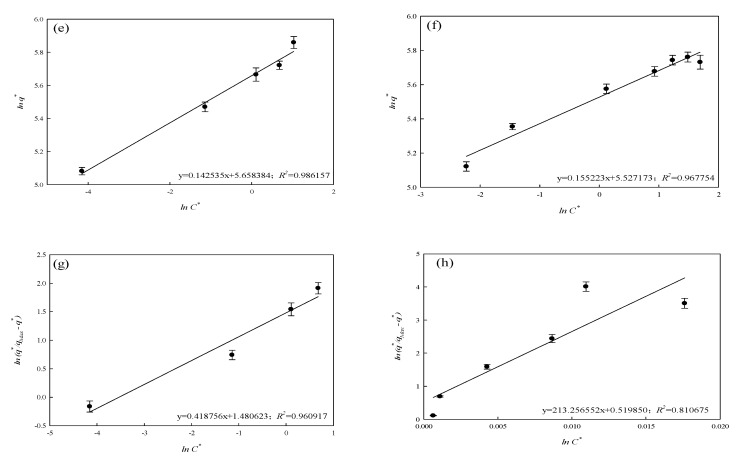
Equilibrium adsorption isotherms for (**a**,**c**,**e**,**g**) AO7 and (**b**,**d**,**f**,**h**) TBO on P-COOH-CS-CEW nanofiber membrane. Equilibrium adsorption isotherms fitted by Langmuir model (**c**,**d**), Freundlich model (**e**,**f**), and Langmuir–Freundlich model (**g**,**h**).

Isothermal adsorption behavior of P-COOH-CS-CEW for AO7 and TBO dyes (initial dye concentration: 0.1–5.0 mg/mL) was investigated, and the results are shown in Figure 8a,b and Table 1. The experiment data were fitted to three equilibrium isotherm models (Langmuir, Freundlich, and Langmuir–Freundlich models) using Equations (1)–(3), respectively [46]:

(1) $$\frac{C^{*}}{q^{*}}=\frac{K_{L}}{q_{max}}+\frac{C^{*}}{q_{max}}$$

(2) $$lnq^{*}=lnK_{F}+\frac{1}{n}lnC^{*}$$

(3) $$ln\frac{q^{*}}{q_{max}-q^{*}}=nlnC^{*}-lnK_{LF}$$

where C* (mg/mL solution) is equilibrium dye concentration in the aqueous phase, q* (mg/g membrane) is the amount of dye on the membrane solid phase, and *q_max_* is the maximum capacity binding of P-COOH-CS-CEW (mg/g). *K_L_* is the dissociation equilibrium constant (mg dye/mL) of the membrane-dye complex fitted by Langmuir model, and the reciprocal *K_L_* value is the equilibrium constant. *K_F_* and 1/*n* values are the adsorption constants in Freundlich model. *K_LF_* represents the dissociation constant (mg/mL) of the complex in Langmuir–Freundlich model, and 1/*n* is the Freundlich coefficient, which represents the characteristic parameter, where 1/*n* = 1, the adsorption conforms to the Langmuir equation. 1/*n* > 1 means that the adsorption phenomenon is a positive synergy; 0 < 1/*n* < 1 is a negative synergy.

The curve fitted by Langmuir model was rearranged into linear form as shown in Figure 8c,d; the fitting of experiment results for both AO7 and TBO dyes yields the high linear coefficient of determination (*R*^2^ = 0.998). However, the experiment results did not fit well to the adsorption behavior described by Freundlich (*R*^2^ = 0.968–0.986) and Langmuir–Freundlich adsorption models (*R*^2^ = 0.813–0.961), as shown in Figure 8e–h, respectively. Based on Langmuir model, the maximum equilibrium adsorption capacity and dissociation constant of AO7 adsorption process by nanofiber membrane were 329.36 mg/g and 0.038 mg/mL, respectively; the equilibrium binding constant was *K_eq_* = 26.32 mL/mg. For the adsorption of TBO dye, the maximum equilibrium binding capacity and dissociation constant were 317.16 mg/g and 0.107 mg/mL, respectively; the equilibrium binding constant (*K_eq_*) is 9.35 mL/mg. The results suggested that binding capacities of P-COOH-CS-CEW nanofiber membranes for both dyes are the same. Nevertheless, for the case of AO7 dye adsorption by P-COOH-CS-CEW nanofiber membranes, a greater value of equilibrium binding constant implies that the protein-modified membrane has a higher binding affinity for AO7. Therefore, the binding strength between AO7 and P-COOH-CS-CEW would be higher. The possible reason is that the electrostatic interaction is the main force involved in the dye adsorption process. In addition, the combination of hydrophobic interaction and hydrogen bond may be present between the dye and P-COOH-CS-CEW nanofiber membrane. Thus, the intermolecular interactions, including electrostatic attraction, Van der Waals interaction, and hydrogen bonding, are expected to take place between P-COOH-CS-CEW nanofiber membrane and the dye.

### 3.5. Kinetic Adsorption Studies

**Figure 9:**
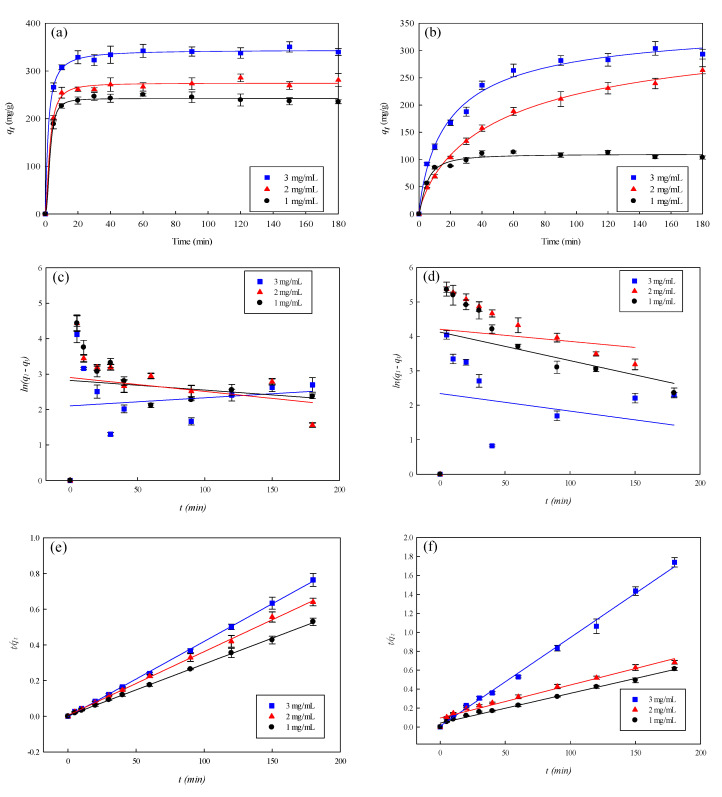
Adsorption rates of P-COOH-CS-CEW nanofiber membrane for (**a**) AO7 and (**b**) TBO dyes. Kinetic adsorption of (**c**) AO7 and (**d**) TBO fitted by pseudo first-order model. Kinetic adsorption of (**e**) AO7 and (**f**) TBO fitted by pseudo second-order model.

Figure 9a–f and Table 2 show the results of the kinetics of dye adsorption by P-COOH-CS-CEW nanofiber membrane at various concentrations of dyes (1–3 mg/mL). It was found that the adsorption of AO7 and TBO ceased after about 30 min (Figure 9a) and 90 min (Figure 9b), respectively. Therefore, P-COOH-CS-CEW nanofiber membrane has a higher selectivity for AO7 dye than TBO dye. When the initial dye concentration increased from 1.0 to 3.0 mg/mL, the concentration of adsorbed AO7 dye increased from 236.57 to 337.75 mg/g; in contrast, the concentration of adsorbed TBO dye increased from 104.49 to 304.33 mg/g. The increase in concentration of adsorbed dyes led to a higher removal rate because of the difference in concentration between liquid and solid (membrane) phase. The drastic concentration difference between adsorbent and liquid phase facilitates the collision between dye molecular and membrane binding site. As CEW proteins are immobilized on the surface of the P-COOH-CS-CEW nanofiber membrane, the adsorption of dye molecules at the outer surface of the membrane was expected.

Two adsorption models, namely, pseudo first- and second-order kinetic models [Equations (4) and (5), respectively] [47], were applied in the study of adsorption kinetics of P-COOH-CS-CEW nanofiber membrane and in the determination of the adsorption rate constant for the cases of AO7 and TBO adsorptions.

(4) $$ln\left(q_{1}-q_{t}\right)=lnq_{1}-k_{1}t$$

(5) $$\left(\frac{t}{q_{t}}\right)=\left(\frac{1}{k_{2}q_{2}^{2}}\right)-\left(\frac{1}{q_{2}}\right)t$$

where *q*_1_, *q*_2_, and *q_t_* represent the binding capacity (mg/g) of nanofiber membrane for dye at any given time *t* and at equilibrium, *t* is the adsorption time*, k*_1_ is the pseudo first-order kinetics rate constant, *k*_2_ is pseudo second-order kinetics rate constant. Table 2 shows the adsorption rate constants derived from the fitting of pseudo first- and second-order kinetic adsorption model, as well as the binding capacity of P-COOH-CS-CEW for AO7 and TBO dyes. The pseudo first-order kinetic adsorption model gave the low *R*^2^ values (<0.0480 for AO7 dye, Figure 9c; <0.0874 for TBO dye, Figure 9d), and therefore it is not suitable for describing the adsorption behavior. Figure 9e,f show the performance of the data fitting by pseudo second-order kinetic model, which gave the high *R*^2^ values (>0.997); pseudo second-order kinetic models are hence suitable for describing the dye adsorption behavior. Moreover, the surface reaction on the nanofiber membrane may be the rate-determining step of dye adsorption. In the case of the pseudo second-order kinetics, the calculated *q_cal_* value for AO7 dye adsorption is 345.12 mg/g, which is quite close to the maximum adsorbed amount of AO7 (333.75 mg/g) as described by Langmuir model. For the adsorption of TBO by P-COOH-CS-CEW nanofiber membrane, the calculated binding capacity (*q_cal_* = 318.01 mg/g) is close to the maximum adsorbed amount of TBO (317.16 mg/g) as described by the Langmuir model.

### 3.6. Desorption Studies

**Figure 10:**
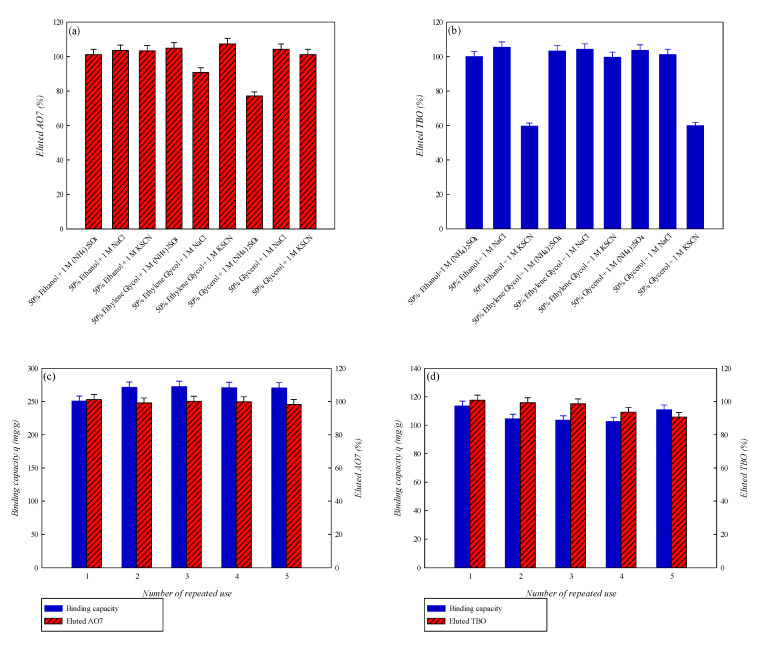
Adsorption–desorption, repeated use, and regenerated binding capacity of P-COOH-CS-CEW nanofiber membrane for the removal of AO7 and TBO dyes: (**a**) Eluted AO7 (%), (**b**) Eluted TBO (%), (**c**) Binding capacity for AO7 after repeated use, and (**d**) Binding capacity for TBO after repeated use.

Studies of adsorption–desorption characteristics are crucial for assessing the regeneration and reusability of the membrane adsorbent. To study the possibility of regenerating P-COOH-CS-CEW nanofiber membrane for multiple rounds of adsorption-desorption, various salts [1 M, NaCl, (NH_4_)_2_SO_4_, and KSCN] dissolved in different organic solvents (50% ethanol, ethylene glycol, and glycerol) were used for desorption. As shown in Figure 10a,b, depending on the combinations of organic solvents and salts as dye-elution reagent, the complete elution of AO7 and TBO dyes from P-COOH-CS-CEW nanofiber membrane can be attained. This implied that the electrostatic or van der Waals forces were present between dye and binding site on the nanofiber membrane. However, there may be other intermolecular forces between the dye and protein on the P-COOH-CS-CEW nanofiber membrane (e.g., hydrophobic and hydrogen bonding). The adsorbed dyes, either AO7 or TBO, can be thoroughly eluted from the P-COOH-CS-CEW nanofiber membrane by 1 M NaCl solution containing 50% ethanol, as shown in Figure 10c,d. Finally, after five adsorption–desorption cycle tests, the binding capacity of P-COOH-CS-CEW nanofiber membrane for both dyes remained at ~97%, indicating that the P-COOH-CS-CEW nanofiber membrane has good potential for the repeated use in dye wastewater treatment.

### 3.7. Remarks on Protein Modified Nanofiber Membrane for Dye Waste Treatment

Dye wastewater has severe negative effects on the environment if it is not pretreated properly. Adsorption technique can be an effective approach for the treatment of dye wastewater. However, the conventional adsorption techniques may have low efficiency in dye removal. To improve the interaction between the dye and P-COOH-CS-CEW nanofiber membrane, natural polysaccharides (i.e., chitosan) and CEW proteins have been coupled onto P-COOH nanofiber membrane with the aim to improve the dye adsorption via the effects of electrostatic attraction, van der Waals force, and hydrogen bonding. These molecular interactions between the hydrophobic group of dye and non-polar side chain of amino acid residue are responsible for the improved adsorption performance. The charge properties of the protein can be adjusted via buffer pH. In this case, the protein molecules immobilized on the P-COOH-CS-CEW nanofiber membrane not only can remove the positively charged dye, but also remove negatively charged dye. The results of this study are compared with other published studies on the adsorption of AO7 [2,48,49,50,51,52] and TBO [8,10,12,14,15,40,47] as shown in Table 3. The removal efficiency of AO7 by P-COOH-CS-CEW nanofiber membrane is higher than that of the conventional adsorbents used in other reported studies. As for the removal of TBO, the performances of nanofiber membranes immobilized with CEW or BSA (i.e., P-COOH-CS-CEW, P-COOH-CEW, P-COOH-BSA) are superior to other conventional adsorbents. From this comparison, it is clear that proteins immobilized on the nanofiber membranes could act as a good adsorbent of dye. However, the binding capacity of P-COOH-CS-CEW nanofiber membrane for TBO was lower than that of P-COOH-BSA or P-COOH-CEW nanofiber membranes. This decrease might be attributed to steric effects generated by the high concentration of chitosan and CEW molecules on the P-COOH-CS-CEW nanofiber membrane. The TBO dye molecules may be unable to approach the binding sites on the CEW protein molecules. As a result, the degree of steric hindrances between the CEW protein molecules increased, resulting in the decrease in the membrane’s binding capacity of the membrane for TBO dye.

The nanofiber membranes functionalized with protein for the removal of ionic dyes from wastewater are currently established at the laboratory scale. There are several challenges in transitioning this technique from the laboratory scale to the commercial applications; for example, the mass production of this nanofiber membrane is restricted by the availability of large-scale electrospinning devices and the difficulty of chemical modification of the membrane. Furthermore, the limited stability and the low reliability of the commercial electrospinning process in the fabrication of uniform and high-quality nanofibers remain the significant concerns that must be addressed. Furthermore, the nanofibers’ limited mechanical stability and reusability hinder their capability to be used in the large-scale water and wastewater treatment. Hence, there is a need for developing advanced technological solutions to improve the physical and chemical performances of nanofibers [53,54,55]. The suggestions for future works are as follows: (1) Further improvement of the stability, mechanical strength, and reusability of nanofiber membranes by means such as adjustment of the compositions of polymer solutions or water-insoluble polymer solutions; (2) Design and application of nanofiber membrane reactor in dynamic adsorption membrane chromatography.

**Table 3:** Comparison of adsorption capacity of various adsorbers for AO7 and TBO dyes.

Type of Adsorbent	*q_max_* (mg/g)	*q_max_* (μmol/g)	Reference
AO7			
P-COOH-CS-CEW Nanofiber membrane	329	939	This work
Magnetic graphene/chitosan	43	122	[2]
Canola stalk	25	72	[49]
Spent brewery grains	30	87	[52]
Untreated sugarcane bagasse	28	80	[56]
*Azolla rongpong*	77	220	[51]
Beech wood sawdust	5	14	[57]
Bottom ash	4	12	[48]
TBO			
P-COOH-CS-CEW Nanofiber membrane	317	1037	This work
P-COOH-BSA Nanofiber membrane	435	1422	[42]
P-COOH-CEW Nanofiber membrane	546	1785	[43]
Mesoporous silica	57	186	[8]
Magnetic multi-walled carbon nanotube	53	174	[12]
Almond shell (*Prunus dulcis*)	73	239	[15]
Water-insoluble starch sulfate	27	88	[47]
Gypsum	28	92	[14]
Turkish zeolite	64	210	[10]
Polysulphone-COOH Nanofiber membrane	116	380	[40]
Silica-iron oxide nanoparticles	37	121	[58]

## 4. Conclusions

This study demonstrates the excellent capability of protein-immobilized nanofiber membranes for adsorbing dyes from the simulated wastewater. In specific, P-COOH-CS-CEW nanofiber membrane can be used to effectively remove both positively charged TBO and negatively charged AO7 dyes from aqueous solutions. The binding between dye and P-COOH-CS-CEW nanofiber membrane was mainly driven by the electrostatic forces. The isothermal adsorption studies showed that the equilibrium adsorption behavior can be described by Langmuir model, while the adsorption kinetic studies showed that the dye adsorption behavior conformed to the pseudo second-order kinetic model. The maximum removal rate of AO7 by P-COOH-CS-CEW nanofiber membrane was achieved within 30 min, recording the maximum amount of dye absorbed (329.36 mg/g). In contrast, the adsorption of TBO by P-COOH-CS-CEW nanofiber membrane took around 90 min to reach an equilibrium, and the amount of TBO adsorbed was as high as 317.16 mg/g. In addition, the repeated uses of P-COOH-CS-CEW nanofiber membrane did not significantly reduce the subsequent binding efficiency of nanofiber membrane for the dyes. Therefore, P-COOH-CS-CEW nanofiber membrane is regarded as a potential high-efficiency adsorbent that can remove cationic and anionic dyes from the contaminated water source.

## Figures and Tables

**Table 1:** Equilibrium and thermodynamic parameters calculated using equilibrium isotherm models for determining the adsorption behavior of P-COOH-CS-CEW nanofiber membrane for AO7 and TBO.

Langmuir			Freundlich			Langmuir–Freundlich		
*q_max,cal_* (mg/mL)	*K_L_* (mg/mL)	*R* ^2^	*n_F_*	*K_F_* (mg/mL)	*R* ^2^	*n_LF_*	*K_LF_* (mg/mL)	*R* ^2^
329.36	0.038	0.998	7.016	286.7	0.986	0.419	4.396	0.961
317.16	0.107	0.998	6.442	251.4	0.968	213.3	1.682	0.811

**Table 2:** Pseudo first-order and pseudo second-order kinetic rate constants, calculated and experiment *q* values for the adsorption of AO7 and TBO dyes onto the P-COOH-CS-CEW nanofiber membrane at different dye concentrations.

Kinetic Model		Pseudo Second-Order Model			Pseudo First-Order Model	
AO7 (mg/mL)	*q_exp_* (mg/g)	*q*_2*,cal*_ (mg/g)	*k*_2_ (g/mg·min)	*R* ^2^	*k*_1_ (1/min)	*R* ^2^
1	250.35	237.03	7.37 × 10^−3^	0.9994	2.03 × 10^−3^	0.0181
2	285.76	280.01	2.50 × 10^−3^	0.9989	3.94 × 10^−3^	0.0430
3	337.75	345.12	2.70 × 10^−3^	0.9995	2.74 × 10^−3^	0.0183
TBO (mg/mL)	*q_exp_* (mg/g)	*q*_2*,cal*_ (mg/g)	*k*_2_ (g/mg·min)	*R* ^2^	*k*_1_ (1/min)	*R* ^2^
1	113.56	106.98	9.06 × 10^−3^	0.9963	5.10 × 10^−3^	0.0536
2	263.85	286.88	1.29 × 10^−4^	0.9725	3.51 × 10^−3^	0.0129
3	304.33	318.01	2.45 × 10^−4^	0.9921	8.28 × 10^−3^	0.0874

## Data Availability

Due to the nature of this research, participants in this study did not agree for their data to be shared publicly, so supporting data are not available.
